# Proliferating Lymphatic Endothelial Cells as a Prognostic Marker in Head and Neck Squamous Cell Carcinoma

**DOI:** 10.3390/ijms23179793

**Published:** 2022-08-29

**Authors:** Cristina Stefania Dumitru, Amalia Raluca Ceausu, Nela Pusa Gaje, Cristian Silviu Suciu, Marius Raica

**Affiliations:** Department of Microscopic Morphology/Histology, Angiogenesis Research Center, “Victor Babes” University of Medicine and Pharmacy, Sq. Eftimie Murgu no. 2, 300041 Timisoara, Timis, Romania

**Keywords:** lymphatic vessels, podoplanin, Ki-67, head and neck squamous cell carcinoma

## Abstract

Podoplanin and Ki-67 are two important markers of cancer progression. The aim of this study is to evaluate double immunostaining for Ki-67 and podoplanin in head and neck squamous cell carcinoma (HNSCC), and to observe the involvement of lymphagiogenesis in tumoral and peritumoral areas, as well as the density of tumor proliferation correlated with histopathological grading. A total of 50 patients with HNSCC were included in this study. We carried out a morphological evaluation of tissue samples, after that, cases were selected for double Ki-67 and podoplanin immunostaining. Podoplanin expression was significantly correlated with histopathological grade (*p* < 0.05; *p* = 0.037) and expression of Ki-67 (*p* < 0.05; *p* = 0.050). A high expression of podoplanin, as well as of the proliferation factor Ki-67, was observed in histopathological grade G3 and the correlation between these (*p* < 0.05; *p* = 0.028), and implication of LMVD and LVI was not significant (LMVD *p* = 0.577; LVI *p* = 0.976). This study demonstrated the importance of double immunolabeling in assessing lymphagiogenesis and tumor proliferation in correlation with histopathological grades in HNSCC.

## 1. Introduction

Head and neck squamous cell carcinomas (HNSCC) accounts to about 6% of all cancers, affecting more than half a million patients worldwide every year, and includes malignant tumors in many parts of this region. The tumor progression and metastasis are associated with tumor microenvironment (TME). The TME contains endothelial, inflammatory, and immune cells, extracellular matrix (ECM), and numerous signal molecules [1]. An important part of these are the endothelial cells responsible for the lymphatic vessels and lymphangiogenesis, because HNSCC tends to extend and metastasize through the lymphatic nodes more than through any other ways. Tumor extension is assumed to spread in a peritumoral manner through existing vessels in the TME, as well as through the invasion of new vessels formed in the primary tumor [2].

Lymphatic endothelial cells (LECs) express specific receptors, such as LYVE-1 (lymphatic vascular endothelial receptor), VEGF-3 (vascular endothelial growth factor), Prox-1 (Prospero Homeobox protein 1), and podoplanin (PDPN). Out of all these, it appears that podoplanin (a transmembrane glycoprotein specifically expressed in lymphatic vessels) has an important role in LEC adhesion, migration, and lymphangiogenesis [3]. In healthy tissue, PDPN is expressed in renal podocytes, alveolar type-1 cells, lymphatic endothelium, skeletal muscles, the placenta, lung, and heart; in the myofibroblasts of the breast and salivary glands, the osteoblasts and mesothelial cells and in the basal layer of the epidermis. The expression of PDPN is high in a number of different cancers, including HNSCC [4].

In head and neck cancer, PDPN is a marker of lymphatic vessels; in predicting cancer progress risk, it was correlated with metastasis, recurrence, and poor clinical prognosis of patients with HNSCC. PDPN involvement in epithelial-to-mesenchymal transitions has been demonstrated. In some studies, podoplanin was observed to be expressed in some hyperplastic and dysplastic lesions adjacent to primary oral cancers, suggesting that podoplanin expression may occur in early oral tumors and may play a role in malignant transformation [5].

Another important marker for HNSCC is Ki-67, a non-histone nuclear protein that actively proliferates in normal and tumor cells [6]. Ki-67 is expressed specifically in the nucleus and is present in proliferating cells during G1 to M phases of the cell cycle. Thus, Ki-67 is an important marker showing the division of tumor cells, but is not a marker of malignancy. Ki-67 expression is significantly correlated with histological grading, and this could indicate a poorer prognosis for the patients [7,8].

The aim of this study is to evaluate a Ki-67 and podoplanin double immunostaining in HNSCC to observe the involvement of lymphatic vessels (LVs) in tumor and peritumoral areas, as well as the density of the tumor proliferation correlated with histopathological grading. We assessed PDPN expression in tumor cells, the presence, morphology and density of lymphatic vessels, and the existence of lymphovascular lesions with tumor invasion. LVs invasion factor was assessed by Ki-67 proliferation expression.

## 2. Results

Tissue samples were graded according to Broder histological criteria: grade G1 (well differentiated) 8% of cases (n = 4), grade G2 (moderately differentiated) 38% (n = 19) of cases, and grade G3 (poorly or undifferentiated) 54% of cases (n = 27). In terms of anatomical report, most squamous cell cancers were present at the laryngeal (n = 30; G1 = 3; G2 = 13; G3 = 14), followed by oropharyngeal (n = 13; G1 = 2; G2 = 4; G3 = 7), nasopharyngeal (n = 3; G3 = 3), nasosinusal (n = 2; G3 = 2), and cutaneous (auricular pavilion, nasal tegument) locations (n = 2; G3 = 2).

**Figure 1:**
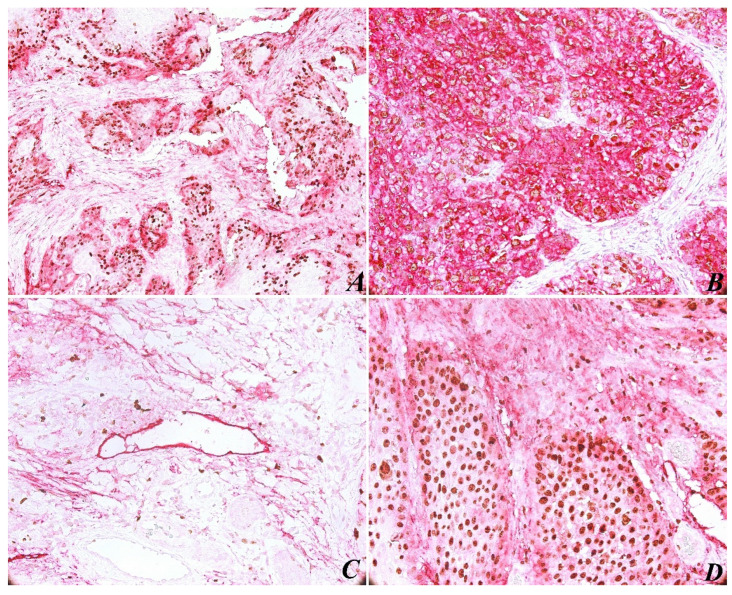
Head and neck squamous cell carcinoma specimens stained with the double immunostaining Ki-67 and podoplanin (purple for podoplanin and brownish for Ki67). High reactivity of podoplanin was present at the periphery of tumor areas, score +2, original magnification ×200 (**A**). Height expression of podoplanin score +3 and Ki-67 score +3 in tumor area, original magnification ×200 (**B**). Peritumoral lymphatic vessel with endothelial wall disrupted by tumor cell invasion, original magnification ×200 (**C**). Podoplanin expression at the periphery of tumor and proliferation index Ki-67 score +3, original magnification ×400 (**D**).

The immunohistochemical evaluation of PDPN expression was identified in almost all cases (with the exception of one case). In most tumors (85%), PDPN was identified in the basal-suprabasal layer of squamous epithelia, with both a cytoplasmic and membranous pattern. As for PDPN immunoexpressing in tumors, high reactivity was present at the periphery of most tumor areas (Figure 1A). As expected, PDPN was highly expressed in cytoplasmic areas of tumor cells (Figure 1B), correlating with high histological grading. The results show a significant correlation between PDPN immunoexpressing and histopathological grading (*p* < 0.05; *p* = 0.037) (Table 1).

The average lymph microvessel density (LMVD) assessment score was calculated using three consecutive fields with original magnification ×200. We found that the average LMVD was higher in the peritumoral area in the G3 grading score (maximum score 13.66 × 200 magnification) than in the intratumoral area (minimum score 0.66 × 200 magnification). From the point of view of LV morphology, intratumoral LVs have small lumen, most often invaded by tumor cells and peritumoral LVs have large, relatively regular lumen with irregular outline. We correlated LMVD with the conventional prognostic element (histological grading of cases), considering the possible prognostic implication of LMVD in HNSCC. We did not obtain a statistically significant correlation with tumor grading (*p* < 0.05; *p* = 0.577), (Table 2), but there was a significant correlation with PDPN score (*p* < 0.05; *p* = 0.007) (Table 3).

Another observation concerned lymphovascular invasion (LVI), which is defined by the identification in the LV lumen of isolated tumor cells, small groups of tumor cells, or by discontinuous wall of the lymphatic vessels by tumor invasion (Figure 1C). In this case, due to Ki-67/PDPN double staining, we found 15 tissue samples with LVI, counting the entire tumor tissue sample. We found no major differences between peritumoral and intratumoral LVD, no significant correlation between LVI and histological grading (*p* < 0.05; *p* = 0.976), and tumor proliferation assessed by Ki-67 expression was not relevant (*p* < 0.05; *p* = 0.413). We believe that such a correlation was not possible because the current stage of the tumor evidence did not show loco-regional lymph node metastasis.

The immunohistochemical evaluation of Ki-67 nuclear staining was observed in all 50 cases. An increased Ki-67 proliferation index was present in 82% of tumor areas (Table 4), and a statistical correlation was found between this and histological grading (*p* < 0.05; *p* = 0.050). In addition, we recorded a statistically significant correlation between Ki-67 expression and PDPN expression (*p* < 0.05; *p* = 0.028). However, an analysis of Ki-67 nuclear labeling and LMVD profile was not significant (*p* < 0.05; *p =* 0.896). In the tumor areas, both peripheral and central cells of the tumor islets were positively immunostained with Ki-67 (Figure 1D). Immunoreactivity for Ki-67 was limited to the nucleus in all samples, and PDPN staining was present in different intensities in cytoplasmic tumor cells.

## 3. Discussion

Despite a high (50.5%) mortality rate of HNSCC and an increased understanding of the mechanisms of carcinogenesis in recent years, the patient survival rate remains less than 50% worldwide [9]. For this reason, molecular studies are essential in the evaluation of tumorigenic aggressiveness factors and in the development of new cancer therapies [10]. In this study, we used double staining markers with podoplanin and the cell proliferation marker Ki-67 to evaluate lymphangiogenesis in head and neck squamous cell cancer tissues. In our analysis, we found significant results for both Ki-67 and Picoplatin expression in association with histopathological tumor grade (podoplanin *p* = 0.0378; Ki-67 *p* = 0.0503).

Lymph node metastases are considered a major prognostic factor in the evolution of HNSCC and represent an important starting point in the therapeutic strategy. The mechanism of tumor dissemination via the lymphatic route is not fully elucidated if this is via lymphangiogenesis or pre-existing lymphatics [4,11]. It has been shown, however, that the structure of lymphatic vessels and their density differ in squamous cell carcinomas, depending on the intratumoral or peritumoral area [3]. In our study, peritumoral lymph vessel density predominated, but a correlation of LMVD and LVI with histopathological grades was not significant (LMVD *p* = 0.577; LVI *p* = 0.976). A predominant statistical correlation between lymphangiogenesis, tumor area, lymph nodes, and histopathological grade has been demonstrated in different studies. Even though peritumoral lymphatic vessels are involved in metastasis, it is not clear whether intratumoral lymphatic vessels are involved in this process, as they are often occluded by tumor cells [12,13]. We consider a limitation of the results in this study which is given by the inclusion of patients in both different histopathological stages of HNSCC and different tumor staging. Thus, statistically important differences in correlation in lymphangiogenesis could be masked by the heterogeneity of cases. On the other hand, LMVD has been highly correlated with podoplanin expression in tumor cells (*p* = 0.007), supporting the important role of podoplanin in the progression, invasion, and metastasis of head and neck tumors. Tumor invasion of lymphatic vessels and tumor emboli can be difficult to detect intratumorally and peritumorally, but double staining facilitated their observation in 7.5% of cases (n = 15).

Regarding the involvement of intratumoral and peritumoral LVs in tumor spread, there are controversial studies in the literature. Thus, animal model studies have demonstrated the inability of tumor vessels to proliferate through tumor compression. On the other hand, studies on human tumors have shown intratumoral involvement of LVs in tumor proliferation and lymph node metastasis to be a poor prognostic indicator [3,9]. Moustakas A et al. explained that the involvement of podoplanin in tumor progression and metastasis could be explained by its increased cell motility and its ability to remodel the actin cytoskeleton of tumor cells [14].

The proliferation index Ki-67 has been shown to be an important marker in HNSCC in numerous studies. Thus, a high expression of Ki-67 could indicate a poorer prognosis for the patients, being associated with a high rate of lymph node metastasis [15,16,17]. In our case, an increased Ki-67 proliferation index was present in 82% of the tumor areas, and there was a statistical correlation between it and histological grading (*p* = 0.050) and with PDPN expression (*p* = 0.028). Most studies have shown the importance of using the Ki-67 marker in squamous cell tumors of the head and neck for prognostic purposes, detection of premalignant lesions, or implications for therapeutic treatments [18,19].

Although intratumoral involvement of LVs in lymph node metastasis with a negative prognostic value has been observed in the literature, these could result from the different methods used to detect intra- and peritumoral LVD. Even though significant progress has been made in understanding the lymphogenetic mechanism by detecting lymphatic endothelial markers, many studies involving Ki-67/podoplanin double staining have not included patients with HNSCC. The results of the current study support the importance of double staining (Ki-67/podoplanin) in determining tumor lymphangiogenesis, increasing the accuracy of diagnosis and prognosis for the patients with HNSCC. Given the short period since the start of the study, it is limited in that a clinical outcome is missing; it is unlikely to assess the survival of patients by applying, for example, the Kaplan–Meier method. The mechanism of podoplanin involvement in these tumorigenic processes needs to be extensively reviewed in HNSCC, necessitating more studies involving lymph node invasion according to anatomical areas of the head and neck. Our results reveal the importance of double staining in the assessment of tumor spread, prognosis, and evaluation of lymph node metastasis, but also as a therapeutic target for HNSCC therapy in future studies.

## 4. Materials and Methods

*Patients and Tissue Samples.* Surgical tissue samples and biopsies were evaluated from 67 patients with tumors from different neck and head areas (larynx, oropharynx, nasopharynx, nasal, sinuses, head skin, laterocervical lymph nodes), but only 50 patients with histopathological diagnosis of squamous cell carcinoma were included in this study. Informed consent was obtained from all subjects involved in the study. The principles of the Declaration of Helsinki were respected, and the study was approved by the Institutional Review Board of Scientific Research Ethic Committee Victor Babeş University of Medicine and Pharmacy Timisoara No.22/September 2019. The tissue samples were fixed in 10% buffered formalin for 24 h and paraffin embedded. After the morphological evaluation and Broder’s system grading, the cases were selected for immunohistochemistry.

*Immunohistochemistry.* The Ki67/podoplanin double immunostaining was applied. The following steps of the immunohistochemical technique were applied: heat-induced epitope retrieval with Bond Epitope Retrieval Solution 2 (Leica Biosystems, Newcastle Ltd., Newcastle upon Tyne, UK) for 20 min, endogenous peroxidase blocking (5 min), incubation with primary antibodies (20 min), and visualization with The Bond Polymer Refine Detection System (for 15 min). The primary antibodies used were Ki-67 (clone MIB-1, monoclonal mouse, anti-human, ready to use, Agilent Technologies Denmark ApS Produktionsvej 42, 2600, Glostrup, Denmark), and podoplanin (clone D2-40, mouse monoclonal, anti-human, ready to use, Agilent Technologies Denmark ApS Produktionsvej 42, 2600, Glostrup, Denmark). The chromogen used was 3.3-diaminobenzidine dihydrochloride. Haematoxylin was used as a counterstain. The chromogen and the counterstain were applied for 10 min. The full immunohistochemical procedure was performed with Bond Max Autostainer (Leica Biosystem).

*Microscopic evaluation.* The PDPN expression was graded as: 0 = no expression was observed in any part of the tumoral area; 1 = positive expression only to the basal layer of the epithelium or <20% staining intensity; 2 = positive expression in the basal and suprabasal layers of the epithelium or 21–50%; 3 = positive expression from the suprabasal layer in two or three areas/more than three areas, or >51%. The Ki-67 proliferation index was rated on a scale of 0 to 3 as: 0 = absence of reaction (regarding the tumor part); 1 = less than 10% positive tumor cells; 2 = 11–50% positive tumor cells; 3 = over 50% positive tumor cells. Scores were based on the examination of the entire section from each biopsy on three microscopic tumoral areas with original magnification ×200. Another evaluation was done on intra- and peritumoral lymphatic vascular density (LVD).

*Data analysis.* Statistical analyses were performed using MedCalc^®^ Statistical Software version 20.015 (MedCalc Software Ltd., Ostend, Belgium; 2021). The results were statistically analyzed using the Chi-squared test and a *p*-value of <0.05 was considered as significant.

## 5. Conclusions

This study demonstrated that Ki-67/podoplanin double immunostaining expression correlated with the histopathological grade of HNSCC, suggesting that these markers are reliable in the clinical use and prognosis of cancer patients.

## Figures and Tables

**Table 1:** Podoplanin score expression in correlation with Broder histological criteria.

PDPN Score	Grading			Total	*p*-Value
	G1	G2	G3		
0	0	1	0	1 (2%)	*p* = 0.037
+1	0	4	2	6 (12%)	
+2	4	10	9	23 (46%)	
+3	0	4	16	20 (40%)	
Total	4 (8%)	19 (38%)	27 (54%)	50	

**Table 2:** Lymph microvessel density (LMVD) correlation with Broder histological criteria.

LMVD	Grading			Total	*p*-Value
	G1	G2	G3		
0	1	6	12	19 (38.0%)	*p* = 0.577
0.66	0	1	0	1 (2.0%)	
1	0	0	1	1 (2.0%)	
1.33	0	0	2	2 (4.0%)	
2	0	3	0	3 (6.0%)	
2.33	1	1	1	3 (6.0%)	
2.66	0	1	1	2 (4.0%)	
3.33	0	1	0	1 (2.0%)	
4	1	1	2	4 (8.0%)	
4.33	1	0	0	1 (2.0%)	
5	0	1	1	2 (4.0%)	
5.33	0	1	0	1 (2.0%)	
6.33	0	1	1	2 (4.0%)	
6.66	0	1	0	1 (2.0%)	
7	0	0	1	1 (2.0%)	
10	0	0	1	1 (2.0%)	
10.66	0	0	1	1 (2.0%)	
12.33	0	1	1	2 (4.0%)	
12.66	0	0	1	1 (2.0%)	
13.66	0	0	1	1 (2.0%)	
Total	4 (8.0%)	19 (38.0%)	27 (54.0%)	50	

**Table 3:** Lymph microvessel density (LMVD) correlation with podoplanin (PDPN) score.

LMVD	PDPN				Total	*p*-Value
	0	1	2	3		
0	0	2	9	8	19 (38.0%)	*p* = 0.007
0.66	1	0	0	0	1 (2.0%)	
1	0	0	0	1	1 (2.0%)	
1.33	0	0	1	1	2 (4.0%)	
2	0	1	2	0	3 (6.0%)	
2.33	0	0	2	1	3 (6.0%)	
2.66	0	0	1	1	2 (4.0%)	
3.33	0	0	1	0	1 (2.0%)	
4	0	0	2	2	4 (8.0%)	
4.33	0	0	1	0	1 (2.0%)	
5	0	0	1	1	2 (4.0%)	
5.33	0	1	0	0	1 (2.0%)	
6.33	0	1	1	0	2 (4.0%)	
6.66	0	1	0	0	1 (2.0%)	
7	0	0	1	0	1 (2.0%)	
10	0	0	1	0	1 (2.0%)	
10.66	0	0	0	1	1 (2.0%)	
12.33	0	0	0	2	2 (4.0%)	
12.66	0	0	0	1	1 (2.0%)	
13.66	0	0	0	1	1 (2.0%)	
Total	1 (2.0%)	6 (12.0%)	23 (46.0%)	20 (40.0%)	50	

**Table 4:** Ki-67 index in correlation with Broder histological criteria.

Ki-67 Score	Grading			Total	*p*-Value
	G1	G2	G3		
0	0	0	0	0	*p* = 0.050
+1	1	6	2	9 (18%)	
+2	0	3	13	16 (32%)	
+3	3	10	12	25 (50%)	
Total	4 (8%)	19 (38%)	27 (54%)	50	

## Data Availability

Not applicable.
